# Localization of HIM-19 in the *C. elegans* germline

**DOI:** 10.17912/micropub.biology.000624

**Published:** 2022-08-10

**Authors:** Baptiste Roelens, Anne M Villeneuve

**Affiliations:** 1 Stanford University School of Medicine, Department of Developmental Biology, Stanford, CA, USA; 2 Stanford University School of Medicine, Departments of Developmental Biology and Genetics, Stanford, CA, USA

## Abstract

A complex series of interconnected events during meiotic prophase creates the physical connections between homologous chromosomes essential to ensure their proper partitioning during the first meiotic division. HIM-19 is an important factor that regulates meiotic prophase progression in *C. elegans* , but its molecular function(s) and localization have remained unclear. We show here that tagged HIM-19 expressed from its endogenous locus exhibits dynamic localization in germ cell nuclei that support its proposed role as a regulator of the CHK-2 protein kinase.

**Figure 1. Localization of HIM-19::3xFLAG in the C. elegans germline:**
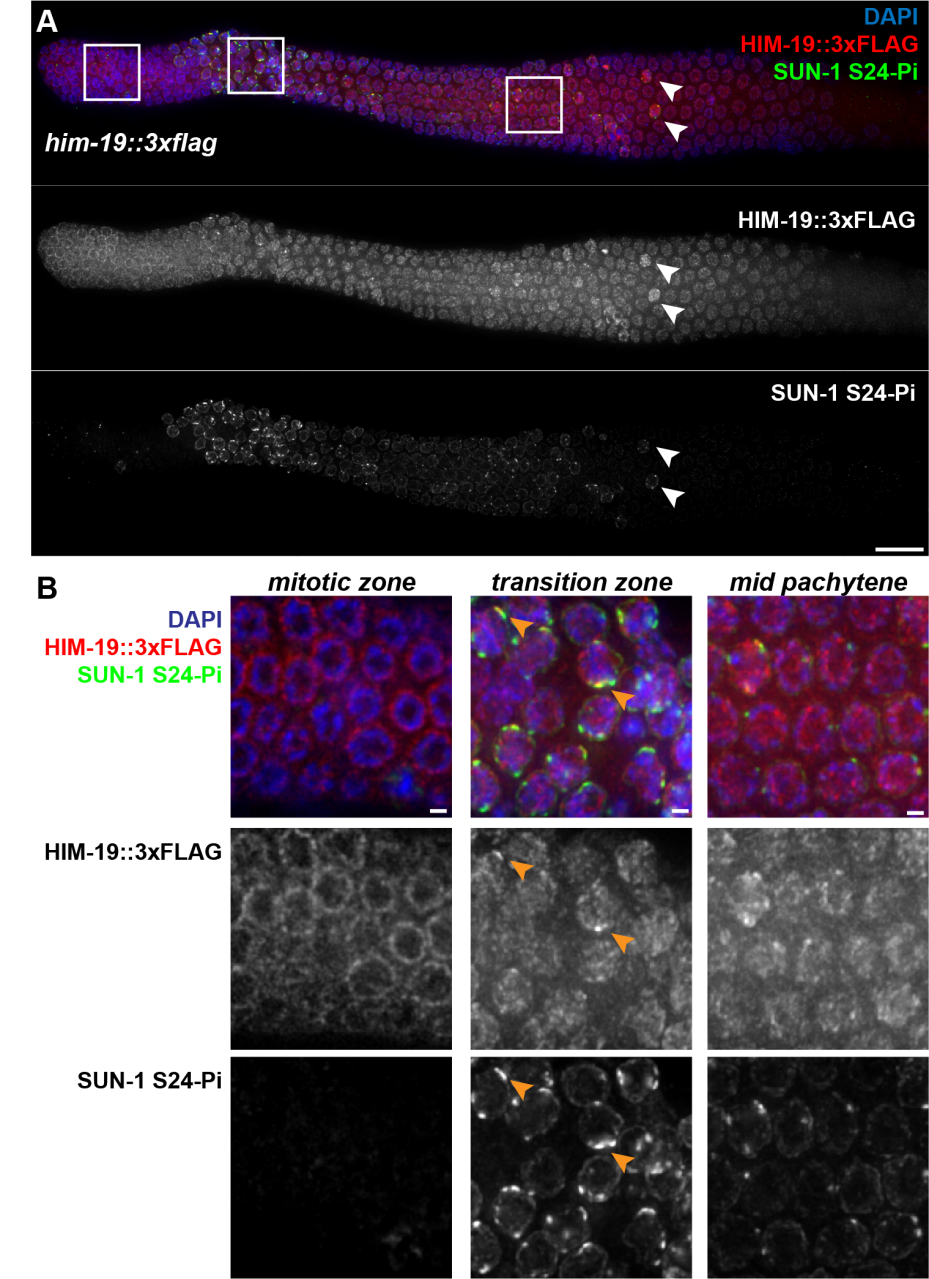
A) Co-immunodetection of HIM-19::3xFLAG and nuclear envelope protein SUN-1 phosphorylated on serine 24 (SUN-1 S24-Pi), a marker of nuclei with active CHK-2, in a whole-mount gonad from phenotypically wild-type worm expressing 3xFLAG-tagged HIM-19 from the endogenous *him-19* locus. White arrowheads indicate “outlier” nuclei with persistent CHK-2 activity; scale bar represents 20µm. B) Zoomed-in images of nuclei from the regions indicated by white boxes in A. Orange arrowheads indicate nuclei in which bright HIM-19::3xFLAG signals coincide with sites of SUN-1 S24-Pi enrichment; scale bars represent 2µm. Images in A and B are maximum intensity projections through the top layer of nuclei of the gonad, except for the mitotic zone image in B, in which a single equatorial slice is depicted to highlight the localization of HIM-19::3xFLAG at the nuclear periphery.

## Description

HIM-19 was previously identified as an important regulator of early meiotic prophase events, as a *him-19* loss of function mutant displayed age-dependent defects in pairing and synapsis (Tang *et al.* , 2010). Similarities between the *chk-2* and the severe *him-19* loss of function phenotypes had suggested that HIM-19 contributes to prophase regulation by promoting/supporting the activity of CHK-2, a master regulator of multiple meiotic prophase events (MacQueen and Villeneuve, 2001; Penkner *et al.* , 2009; Tang *et al.* , 2010). Identification of HIM-19 among the top hits in a recently-reported CHK-2::HA IP/MS analysis further supports a functional connection between HIM-19 and CHK-2 (Baudrimont *et al.* , 2022).

We generated a strain expressing a 3xFLAG-tagged version of HIM-19 and analyzed the localization of HIM-19::3xFLAG in the *C. elegans* hermaphrodite germline. We found that HIM-19 is expressed and enriched in nuclei throughout the gonad, and that its subnuclear localization changes as germ cells develop (Figure 1A, B). In the premeiotic region of the germ line (mitotic zone, B) HIM-19 is concentrated at the nuclear periphery where CHK-2 is enriched at this stage (Baudrimont *et al.* , 2022). Upon entry into meiotic prophase, HIM-19 is released from the nuclear periphery and is detected inside the nucleus; HIM-19 is also occasionally enriched in a few nuclear envelope (NE) associated patches (panel B, transition zone) that correspond to positions of enrichment of CHK-2 and SUN-1 S24-Pi, a marker of CHK-2 activity, at sites where chromosomes are anchored to the nuclear envelope to promote pairing (Penkner *et al.* , 2009; Kim *et al.* , 2015). HIM-19 continues to be detected in the nucleoplasm of germ cells throughout the pachytene zone. Further, we note that high levels of HIM-19 are detected in occasional “outlier” nuclei exhibiting high levels of CHK-2 activity outside the main CHK-2 active zone of the gonad (white arrowheads, Figure 1A). Importantly, as NE-associated SUN-1 S24-Pi patches appeared with normal timing upon meiotic entry in the HIM-19::3xFLAG-expressing strain and we did not observe any defects in meiotic progression (assessed by the length of the SUN-1 S24-Pi zone), number of DAPI-stained bodies in diakinesis oocytes, or incidence of male self progeny, we infer that the 3xFLAG-tagged version of HIM-19 is functional. In conclusion, HIM-19 localization mirrors the previously described localization of CHK-2 in both the premeiotic and meiotic prophase zones, consistent with the proposed role of HIM-19 as a regulator of CHK-2 function.

## Methods

CRISPR mediated genome editing:

Genome editing was performed as described in (Arribere *et al.* , 2014). In short, ribonucleoprotein complexes were assembled by mixing purified Cas9 (PNA Bio) with tracrRNA and sgRNA (Horizon Discovery) directed against *him-19* Ct (ACAAGGCGAGAATTtgaatt) and *dpy-10* . Single stranded repair template (ultramers from Integrated DNA technology) with the *dpy-10(cn64)* and *him-19::3xflag* (GCCCGTTGATCACCGTTTTCAATAAAGATCGTGTGTACAAGGCGAGAATTGGAGGTGACTATAAAGATCACGACGGAGATTACAAGGACCATGATATCGACTACAAGGACGACGACGACAAGGGATGAatttggcagcatatttggcagcagcatccgtatttaatttccattgt) sequences were added and the mix was injected into N2 worms. Individual roller progeny from injected hermaphrodites were singled out and tested for the presence of the *him-19::3xflag* edit by PCR using primers oBR989 (GGGTGCCCGTTGATCACCGTTT) and oBR990 (gaattgcaagcgcgctccagtg).

Cytology: Immunofluorescent detection of HIM-19::3xFLAG and SUN-1 S24-Pi was performed as described in (Martinez-Perez and Villeneuve, 2005) using a mouse monoclonal anti-FLAG (M2, Sigma-Aldrich) and a guinea-pig anti SUN-1 S24-Pi (Penkner *et al.* , 2009). A detailed step by step protocol can be found at: https://dx.doi.org/10.17504/protocols.io.xrgfm3w.

## Reagents

Strains:

**Table:** 

Strain	Genotype	Available from
N2	*Caenorhabditis elegans*	CGC
AV1111	*him-19(me131 [him-19::3xflag])* I	AV lab, will be deposited in CGC
